# Resveratrol as Inducer of Autophagy, Pro-Survival, and Anti-Inflammatory Stimuli in Cultured Human RPE Cells

**DOI:** 10.3390/ijms21030813

**Published:** 2020-01-27

**Authors:** Natasha Josifovska, Réka Albert, Richárd Nagymihály, Lyubomyr Lytvynchuk, Morten C. Moe, Kai Kaarniranta, Zoltán J. Veréb, Goran Petrovski

**Affiliations:** 1 Department of Ophthalmology, Center for Eye Research, Oslo University Hospital and University of Oslo, 0450 Oslo, Norway; natasa_josifovska@yahoo.com (N.J.); enmihaly@gmail.com (R.N.); m.c.moe@medisin.uio.no (M.C.M.); 2 Department of Ophthalmology, Faculty of Medicine, University of Szeged, 6720 Szeged, Hungary; albertreka86@gmail.com (R.A.); Lyubomyr.Lytvynchuk@augen.med.uni-giessen.de (L.L.); jzvereb@gmail.com (Z.J.V.); 3 Department of Ophthalmology, Justus-Liebig-University Giessen, Eye Clinic, University Hospital Giessen, 35390 Giessen, Germany; 4 Department of Ophthalmology, Institute of Clinical Medicine, University of Eastern Finland and Kuopio University Hospital, 70210 Kuopio, Finland

**Keywords:** resveratrol, retinal pigment epithelium, autophagy, proteasomal inhibition, survival, inflammation, protein array, age-related macular degeneration

## Abstract

**Purpose:** To investigate the mechanism by which resveratrol acts upon retinal pigment epithelial (RPE) cells and to characterize its effect upon autophagy, survival, and inflammation, with consequent implications to treatment for age-related macular degeneration (AMD). Methods: Cultured ARPE-19 cells were exposed to 10 and 50 μM resveratrol. Cell survival/death was determined by annexin-FITC/propidium iodide using flow cytometry, while autophagy was studied by detecting autophagic vacuoles formation (acridine orange and transmission electron microscopy), as well as LC3II/I ratio and p62 expression by Western blot. In addition, time-lapse confocal microscopy of a pDENDRA-LC3 expression vector was performed to detect autophagy in transfected ARPE-19 cells under the different treatment conditions. Inhibition of proteasomal and autophagy-lysosomal fusion was carried out by MG-132 and chloroquine, respectively, while induction of autophagy was achieved by rapamycin treatment. Detection of secreted cytokines by ARPE-19 cells using Human XL Cytokine Array was performed under oxidative stress (H_2_O_2_) and resveratrol treatments, respectively. Results: Resveratrol induced autophagy in ARPE-19 cells as determined by augmented presence of autophagic vacuoles, increased LC3II/I ratio and decreased p62 expression, as well as time-lapse confocal microscopy using pDENDRA-LC3 expression vector. Resveratrol acted similarly to proteasomal inhibition and downstream of mammalian target of rapamycin (mTOR), since upstream inhibition of autophagy by 3-methyladenine could not inhibit autophagy in ARPE-19 cells. Co-treatmeant by rapamycin and/or proteasome inhibition showed no additive effect upon autophagy induction. ARPE-19 cells treated by resveratrol showed lower cell death rate compared to untreated controls. Resveratrol induced a specific anti-inflammatory response in ARPE-19 cells. Conclusions: Resveratrol can induce autophagy, pro-survival, and anti-inflammatory stimuli in ARPE-19 cells, properties which could be plausible to formulate future treatment modalities for AMD.

## 1. Introduction

The leading cause of blindness in the elderly in developed countries is age-related macular degeneration (AMD). Many risk factors have been associated with the development and progression of the disease, most importantly aging, although other factors, such as inflammation, oxidative stress, and genetic predisposition have been strongly linked [1].

Characteristic of the disease is the deterioration of the retinal pigment epithelium (RPE) and photoreceptors. The exact cause for this is still to be elucidated, as the underlying mechanism is not clear yet, however, apoptosis, autophagy, and necroptosis are believed to be involved. Autophagy is associated to apoptosis and necroptosis, as well as AMD, while the interplay of the different pathomechanisms of the disease need further clarification [2]. Autophagy as a self-digestive cellular mechanism is involved in the clearance of long-lived cytoplasmic proteins under conditions of stress, starvation and proteasomal inhibition. Proper clearance of such cellular debris is important for maintaining cell survival and vitality of cells and tissues in general. Possibly, autophagy contributes to the turnover and regulation of proteins responsible for countering oxidative stress, such as NFE2L2, that is known to interact with p62, the latter being an important autophagic protein [1,2,3].

The RPE is burdened by an increasing accumulation of debris throughout aging; debris clearance by the autophagolysosomal pathway or its blockage have been of great interest lately, considering their potential in treating AMD [2]. In the early stages of the disease, abnormalities in the RPE and Bruch’s membrane are present and are followed by further degeneration as the disease progresses [4]. The accumulation of damaged proteins in RPE cells is considered a contributing factor for RPE dysfunction in AMD [5].

Impaired or insufficient autophagy activity in aged cells is involved in many diseases, including inflammatory-, neurodegenerative-, heart-diseases and cancer [6]. Autophagy plays a beneficial role in several ocular cell types such as the maintenance of eye’s normal physiology, and maintenance of the photoreceptor outer segment turnover, cellular stress protection and melanin degradation in RPEs [7,8].

Resveratrol is a polyphenolic compound which was initially used in cancer therapy, and has shown beneficial effects against most degenerative and cardiovascular diseases. The good effects of resveratrol have been partially linked to its autophagy-inducing action, as well as its anti-angiogenic properties. Its concentration-dependent differing actions include induction of heat-shock and anti-oxidant proteins, as well as anti-angiogenic (anti-vascular endothelial growth factor (VEGF)) properties [9].

The precise molecular mechanism for the beneficial effects of resveratrol still remains unclear. There are many studies investigating the effects of resveratrol on the eye and its related disorders. Similar to its effects in other diseases, in the eye, resveratrol affects oxidative stress, apoptotosis, tumorogenesis, inflammation, angiogenesis, and vasodilatation [10]. Resveratrol is a very potent inhibitor of intracellular reactive oxygen species (ROS) in RPE cells under oxidative stress [11], and it has been reported to reduce oxidation and cell proliferation by inhibiting extracellular signal regulated kinase (ERK) in these cells [12]. The anti-apoptotic activity of resveratrol is important in the prevention of neurodegenerative diseases including AMD [10].

Exploring the autophagy-, survival- and inflammation-related properties of resveratrol in human RPE cells would thus give an insight into its possible beneficial effect in treating AMD. Resvega, a commercialized product containing resveratrol, has been shown to promote autophagy through upregulation of autophagic flux and autolysosome formation, thus protecting cultured cells [13]. In addition to Resvega, Longevinex is another, currently available over-the-counter drug or complex, both containing resveratrol as the core element that have been tested and used in human AMD trials with added benefits [14]. However, the mechanism by which resveratrol and these drugs act upon RPE cells has not been fully elucidated.

## 2. Results

**Figure 1:**
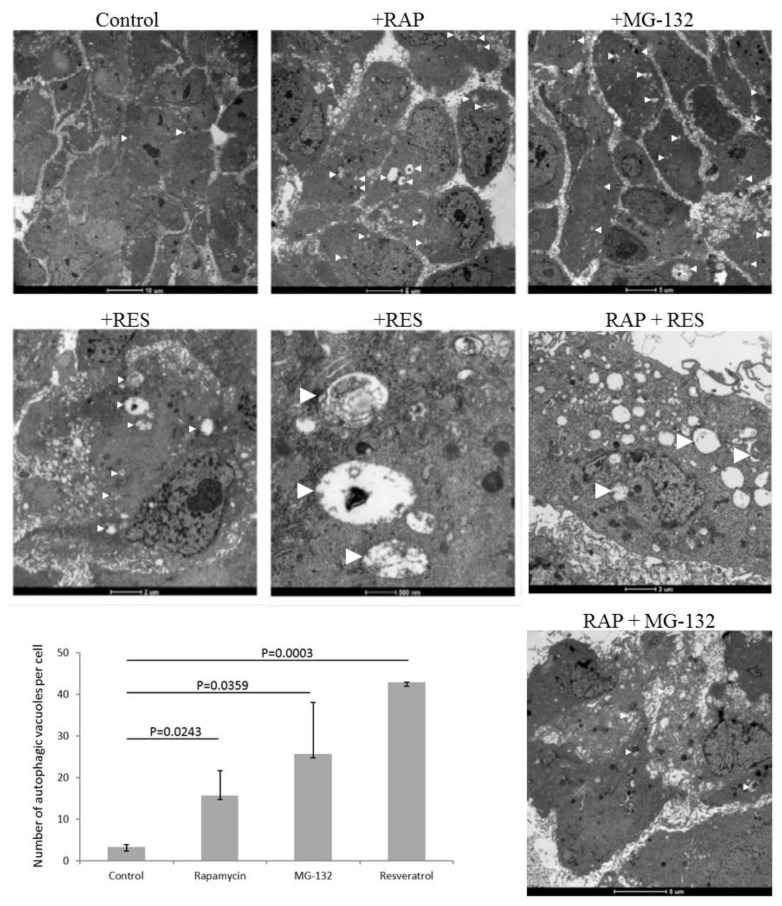
ARPE-19 cell treated by autophagy inducer rapamycin (RAP, 100 nM), proteasome inhibitor MG-132 (100 nM) and resveratrol (RES, 10 µM) over 24 h. Transmission electron microscopy is shown of ARPE-19 cells under different treatment modalities. (Bars on the upper panel from left to right: 10 µm, 5 µm, and 5 µm; middle panel from left to right: 2 µm, 500 nm, and 2 µm; lower panel: 5 µm). The number of autophagic vacuoles per cell is shown for the different treatment conditions. Two-sample *t*-test was used for the analysis.

ARPE-19 cells undergo induced autophagy by rapamycin and MG-132 treatment as shown by enhanced presence of autophagic vacuoles (number of autophagic vacuoles per cell for untreated cells was 3.3 ± 0.6; for rapamycin treated: 15.7 ± 6.0 (*p* = 0.0243); for MG-132 treated: 25.7 ± 12.4 (*p* = 0.0359)) by TEM. Similarly, resveratrol induces autophagy in ARPE-19 cells (number of autophagic vacuoles per cell was 43.0 ± 0.0 (*p* = 0.0003)) (Figure 1). The size of the autophagic vacuoles for the untreated ARPE-19 cells was 748.4 ± 538.4 × 10^3^ nm^2^, which increased under rapamycin treatment (716.0 ± 888.6 × 4 × 10^3^ nm^2^), and decreased under MG-132 (174.1 ± 42.1) and resveratrol (166.4 ± 0.0) treatment. Moreover, co-treatment of rapamycin and resveratrol or MG-132 further enhanced the presence of autophagic vacuoles in the cells.

**Figure 2:**
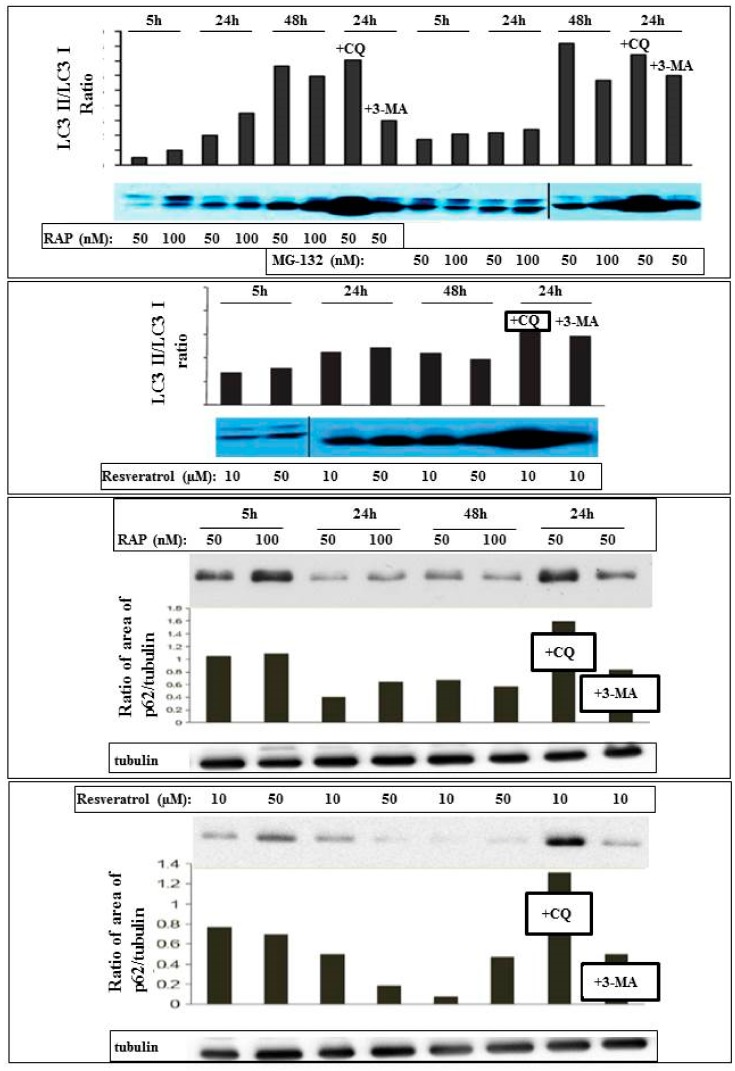
Detection and quantification of autophagy in ARPE-19 cells using Western blot analysis. The cells were treated in a time- and concentration-dependent manner by the autophagy inducer rapamycin (RAP), proteasomal inhibitor MG-132, and Resveratrol. The LC3II/I ratio and expression of p62 are shown, as well as the effect of the autophagosome-lysosome fusion inhibitor chloroquine (CQ) and the upstream inhibitor of autophagy 3-methyladenine (3MA).

The autophagy inducing effect of resveratrol could be further confirmed by increased LC3II/I ratio and decreased p62 expression. This induction was similar to that caused by rapamycin and proteasomal inhibition treatment with MG-132 (Figure 2).

Resveratrol acts similarly to proteasomal inhibition and downstream of mammalian target of rapamycin (mTOR), since upstream inhibition of autophagy with 3-methyladenine does not inhibit autophagy in ARPE-19 cells. Inhibition of the autophagosomal-lysosomal fusion by chloroquine showed presence of autophagic flux, since the ratio of LC3II/I increased with all treatment modalities at the tested time-point (24 h). Co-treatmeant by rapamycin and/or proteasome inhibition showed no additive effect upon autophagy induction.

**Figure 3:**
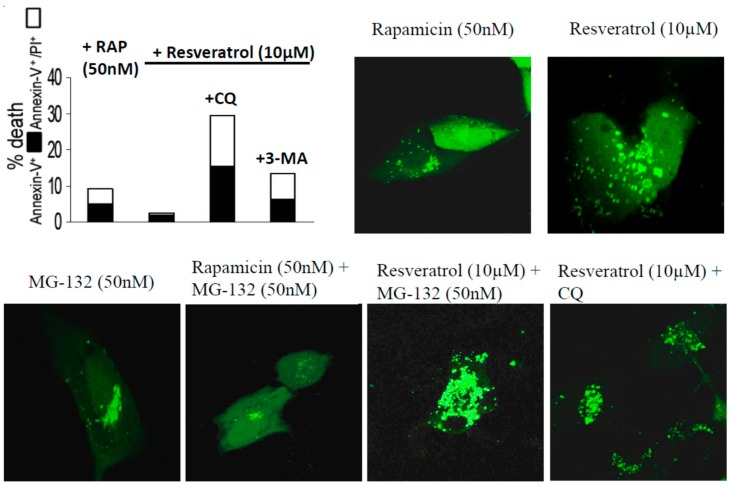
ARPE-19 cells treated by rapamycin (RAP), resveratrol, MG-132, and chloroquine (CQ). The LC3 expression is shown in green using the pDENDRA-LC2 expression vector. Cell death analysis is carried out using Annexin-FITC/propidium iodide expression under the difference treatments.

The autophagy inducing effect of resveratrol, rapamycin and MG-132 on ARPE-19 cells was further confirmed and visualized by time-lapse confocal microscopy using pDENDRA-LC3 expression vector (Figure 3 and Supplementary Materials Video S1 Resveratrol (10 µM) + CQ treatment). ARPE-19 cells treated by resveratrol showed lower cell death rate compared to untreated controls (annexin-V^+^/annexin-V^+^PI^+^: 1.2%/3.5%). The cell death was enhanced under chloroquine and 3-methyladenine treatment (Figure 3).

**Figure 4:**
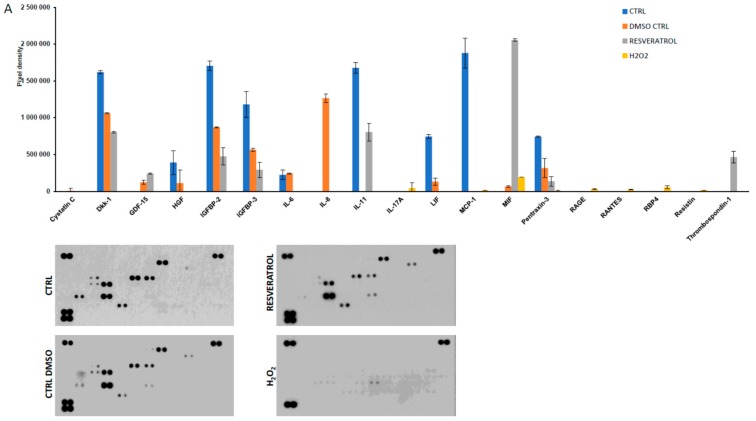

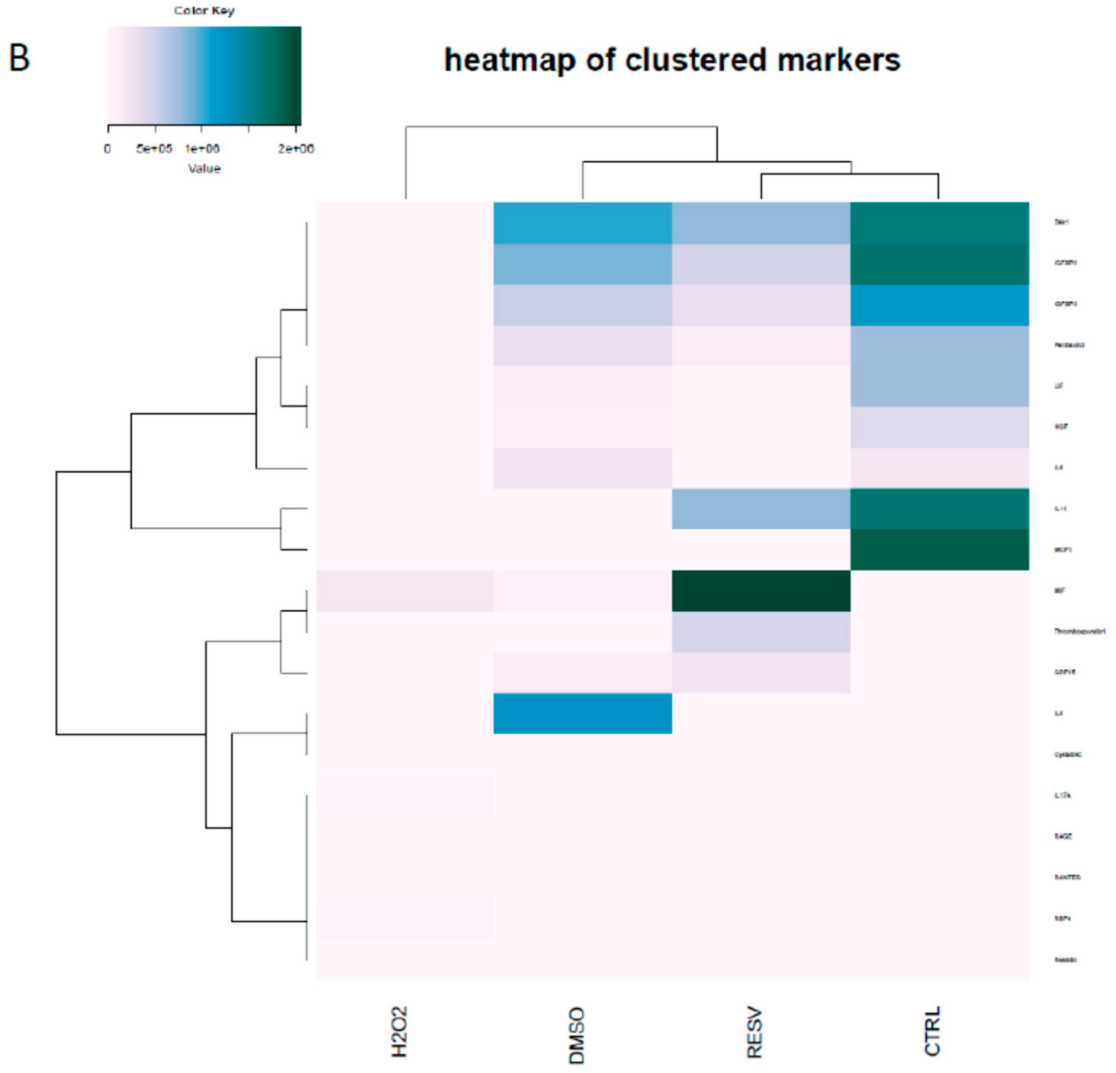
Protein secretome of ARPE-19 cells treated by oxidative stress inducer hydrogen peroxide (H_2_O_2_) and resveratrol (**A**) and hierarchical clustering of markers on a heatmap (**B**).

The secretion of 105 markers (cytokines and inflammation-related factors) were screened from the supernatants of the ARPE-19 cells upon treatment with H_2_O_2_, resveratrol, DMSO, or left untreated (Figure 4). Cystatin-C could be detected at low level only in the DMSO control samples. Interleukin 8 (IL-8 pixel density 1264079 ± 58769, mean ± SD) expressed at a significantly higher level in DMSO treated samples compared to the other setup. Dickkopf-related protein 1 (Dkk-1) was decreased upon DMSO (1060695 ± 4745) and resveratrol (802648 ± 7748) treatments compared to untreated control (1619394 ± 22758). A similar pattern was observed for Insulin-like growth factor-binding protein 2 and 3 (IGFBP2 and IGFBP3) and Pentraxin (also widely known as *TNF*-inducible gene 14 protein/TSG-14), respectively. Receptor for Advanced Glycation Endproducts (RAGE, 30609 ± 1954), Chemokine (C-C motif) ligand 5 (CCL5/Rantes, 24127 ± 2662), Retinol binding protein 4 (RBP4, 55416 ± 23218), adipose tissue-specific secretory factor (Resistin, 15724 ± 689) and Interleukin-17A (IL-17A, 44257 ± 69321) could be detected just when cells were exposed to H_2_O_2_. The presence of resveratrol elevated the amount of secreted growth/differentiation factor 15 (GDF15), also known as macrophage inhibitory cytokine-1/MIC-1; 238983 ± 6539), Thrombospondin-1 (TSP-1, 465751 ± 79848), and macrophage migration inhibitory factor (MIF 2057372 ± 19331) in the cell culture media. Interestingly resveratrol moderated the secreted interleukin-11 (IL-11 805249 ± 120882) level compared to untreated control (1677339 ± 69313). Interleukin-6 (IL-6), Hepatocyte growth factor (HGF) and Leukemia inhibitory factor (LIF) could only be detected in the untreated and DMSO treated control samples.

Hierarchical clustering of secreted cytokines based on their pixel density level clearly separated the H_2_O_2_ treated group from the other experimental subsets. Within the other cluster the cytokine pattern of resveratrol treated cells was similar to the untreated controls, as they formed an individual group within the cluster.

Different levels of the secreted cytokines in H_2_O_2_, DMSO, resveratrol and control ARPE-19 cells are based on the analytes pixel density. The cluster analysis and dendrogram show the difference between the treatments. Blue and white colors indicate high and low pixel density, respectively.

## 3. Discussion

Resveratrol is one of the most studied polyphenols, which is a naturally occurring compound found in at least 72 plant species relevant to human diet such as grapes, peanuts, blueberry, and raspberry. It is a molecule of great interest to science and medicine, due to its beneficial anti-proliferative, anti-oxidative, anti-inflammatory, anti-angiogenic, and anti-metastatic effects in many different cell lines [15,16,17,18]. Resveratrol is widely used in ophthalmology in both, clinical and experimental studies using animal models and in vitro culture models [19]. It has been shown to get rapidly absorbed, both in vivo, in human studies, and in vitro, in cell culture studies with no marked toxicity reported [11,20]. Resveratrol can penetrate the blood-brain and blood-ocular (retinal- and aqueous) barriers, and it has been detected in the intraocular fluids after oral administration [5,21,22].

There have only been few studies working to detect the concentration of resveratrol in human tissues, and while micromolar concentrations can protect against lens epithelial cell apoptosis in diabetic cataracts in rats; furthermore, such concentrations could provide protection against inflammatory ocular surface diseases such as dry eye and severe allergies, and inhibition of the proliferation of retinoblastoma cells; the nanomolar concentrations of resveratrol necessary for effective treatment of the human eyes have, however, not been demonstrated yet [5].

Resveratrol can inhibit the mammalian target of rapamycin (mTOR) that induces autophagy, however, a concentration for achieving such an autophagy-inducing effect in vivo needs to be defined yet [7,23]. Small amounts of stress induced by resveratrol can further induce autophagy and generate a survival signal; at the same time, autophagy induced by higher doses of resveratrol can serve as a death signal, if the amount of stress is large enough to induce cytotoxicity in the cells [7,24].

Resveratrol-induced autophagy in ARPE-19 cells as shown by increased autolysosome formation and autophagy flux, as well as change in the LC3 and p62 protein levels [13] in our study could be further confirmed by TEM. ARPE-19 cells have also been used to show the anti-inflammatory potential of resveratrol through the decrease of IL-6 and IL-8 expression, as well as to show its anti-oxidative properties by exhibiting a cytoprotective effects against the enhancement of IL-8, MCP-1, and VEGF secretion and anti-angiogenic potential by the inhibition of VEGF-A secretion [21,25]. Our results are in agreement with these findings and expand upon the repertoire of other inflammation- and angiogenesis-related factors attenuated by resveratrol (e.g., DKK-1; HGF; IGFBP-2 and 3; IL-11 and 17A; Pentraxin-3). Inversely, resveratrol attenuated factors involved in macrophage inhibition or anti-inflammation (e.g., TSP-1; MIF).

Autophagy can be stimulated by a number of stimulants such as changes in the environmental (e.g., nutrient deprivation, hormonal stimuli) as well as other factors like oxidative stress. One convenient way to experimentally increase autophagy is to treat cells or organisms by the inducer of autophagy-rapamycin. The latter is a lipophilic macrolide antibiotic and a negative regulator of mTOR, found to induce autophagy in many species [26]. Autophagy is involved in the pathogenesis of AMD and its impairment can contribute to the progression of the disease due to failed removal of damaged organelles. Therefore, upregulating autophagy may provide an alternative therapeutic strategy to slow or stop AMD progression [6]. Our study showed that induced autophagy in the ARPE-19 cells by rapamycin and MG-132 is similar to the effect achieved by resveratrol.

RPE cells exposed to lethal concentrations of H_2_O_2_ have been rescued from cell death by improved autophagic efficiency stimulated by rapamycin [6]. Its inhibition of the mTOR pathway could prevent the replicative senescence in cultured RPE cells, while in mouse models of retinal degeneration, rapamycin could prevent photoreceptor dysfunction [27]. It has been shown that rapamycin can also improve the rod photoreceptor cell survival and function in the light damage model, but it can reduce the cone-mediated retinal function in albino mice [26].

Chloroquine (CQ) a lysosomal inhibitor has been shown to reverse autophagy by accumulating in lysosomes, disturbing the vacuolar H^+^ATPase responsible for lysosomal acidification and blockage of autophagy [28,29,30]. CQ can block the fusion of AVs with lysosomes and degradation of autophagic proteins in ARPE-19 cells, which can lead to increased levels of LC3-II [31]. Our Western blot analysis showed presence of autophagic flux upon inhibition of the autophagosomal-lysosomal fusion by CQ, and this could be confirmed by the increased LC3 II/I ratio with all treatment modalities at the tested time-point (24 h).

Overall, we could show resveratrol to be able to induce autophagy, pro-survival and anti-inflammatory stimuli in ARPE-19 cells, properties which may be plausible to formulate future treatment modalities for AMD.

## 4. Materials and Methods

### 4.1. Cell Culture and Treatments

Dulbecco’s modified Eagle’s medium (DMEM), resveratrol, MG-132, chloroquine, and 3-methyladenine were purchased from Sigma (Steinheim, Germany); plastic tissue culture flasks were purchased from TPP (Trasadingen, Switzerland). ARPE-19 human retina pigment epithelial cells were kindly provided by Prof. Stephen Moss, (UCL, London, UK) and were cultured at 37 °C under 5% CO_2_ in a 1:1 mixture of DMEM and Nutrient Mixture F12 Medium supplemented with 10% fetal calf serum (FCS) [32]. Cells were detached from the substrate using trypsin/EDTA (0.05:0.02%). Before resveratrol treatment, cells were plated over a 24-hr period and treated by 10 µM resveratrol for 24 h before harvesting. Table 1 shows the different treatment modalities used in the study.

### 4.2. Assays of Cell Death and Autophagy

Cell death was assessed by the Annexin-V-fluorescein isothio-cyanate Apoptosis Detection Kit (MBL, Woburn, MA, USA) according to manufacturer’s recommendations; proportion of stained Annexin-V^+^ and Annexin-V^+^PI^+^ cells was determined by fluorescence activated cell sorter (FACS) analysis on BD Bioscience flow cytometer. Autophagy was assessed by detection of AVs using acridine orange staining or live confocal microscopy on pDENDRA-LC3 transfected cells [33,34]. Whenever inhibition of autophagy and autophago-lysosomal fusion was carried out with 3-methyladenine (3-MA) and chloroquine, respectively, or induction of autophagy with rapamycin took place, the treatment preceded peaking of autophagy by 1 day.

### 4.3. Transmission Electron Microscopy (TEM)

Samples were fixed in 0.1 M sodium cacodylate-buffered, pH 7.4, and 2.5% glutaraldehyde solution for 2 h and then rinsed (three times, 10 min) in 0.1 M sodium cacodylate buffer, pH 7.4, and 7.5% saccharose and postfixed in 1% OsO_4_ solution for 1 h. After dehydration in an ethanol gradient (70% ethanol (20 min), 96% ethanol (20 min), 100% ethanol (two times, 20 min)), samples were embedded in Durcupan ACM. Ultrathin sections were stained with uranyl acetate and lead citrate. Sections were examined in a Philips CM 10 microscope (Philips Electronic Instruments, Mahwah, NJ, USA) at 80 kV. Quantification of the number and size of the autophagic vacuoles per cell was performed using ImageJ freeware (NIH, USA) and its regions of interest and calibration method.

### 4.4. Antibodies and Immunoblotting

Anti-LC3 polyclonal antibody (Novus Biologicals, Centennial, CO, USA, NB100-2220) was used for detecting autophagy. Cell lysates were separated on NuPAGE 12% Bis–Tris polyacrylamide gel (Invitrogen, Carlsbad, CA, USA) and transferred to Immobilon-P Transfer Membrane (Millipore, Bedford, MA, USA; pore size 0.45 µm). Membranes were blocked in Tris-buffered saline containing 0.05% Tween-20 (TBS-T) and 5% nonfat dry milk (BioRad, Hercules, CA, USA) for 1 h, probed overnight at 4 °C with primary rat anti-LC3 polyclonal antibody in TBS-T containing 1% nonfat dry milk and incubated for 1 h with rabbit anti-rat peroxidase-conjugated secondary antibody (DAKO, Glostrup, Denmark) at room temperature. Peroxidase activity was detected with SuperSignal West Femto Maximum Sensitivity Chemiluminescent Substrate (Pierce, Rockford, IL, USA) using a Lumi-Imager (Roche Diagnostics, Mannheim, Germany).

### 4.5. Detection of Secreted Cytokines by Protein Array

Supernatants of the cell cultures treated by H_2_O_2_, DMSO, or resveratrol were collected, centrifuged and stored frozen until cytokine detection. The secreted factors were determined by Human XL Cytokine Array (Proteome Profiler, R&D Systems, Minneapolis, MN, USA) according to the manufacturers’ protocol and the pixel density in each spot of the membranes was measured by ImageJ software and results were analyzed by hierarchical clustering. Hierarchical clustering based on the pixel density levels was performed by R software [35]. Full list of analytes is found in the Supplementary Materials Table S1.

### 4.6. Statistical Analysis

Each experiment was performed at least three times, and each sample was tested in triplicates. Statistica 7.0 software (StatSoft IncTulsa, OK, USA) was used for the statistical analyses. Statistically significant difference between the two groups (resveratrol treated RPE cells versus untreated) was determined with paired student t-test, and a value of *p* < 0.05 was considered significant. Data are expressed as mean ± SD or SEM. 

### 4.7. Sample and Data Availability

Samples of the compounds are available from the authors. All data in the manuscript will be publicly made available upon acceptance.

## Figures and Tables

**Table 1:** Treatment protocols used in the study.

	5 h		24 h		48 h	
**Rapamycin (RAP)**	50 nM	100 nM	50 nM	100 nM	50 nM	100 nM
**Rapamycin + CQ**			50 nM + 50 μM CQ			
**Rapamycin + 3MA**			50 nM + 10 mM 3-MA			
**MG-132**	50 nM	100 nM	50 nM	100 nM	50 nM	100 nM
**MG-132 + Chloroquine**			50 nM + 50 μM CQ			
**MG-132 + 3-MA**			50 nM + 10 mM 3-MA			
**Rapamycin + MG-132**	50 nM 50 nM	100 nM 100 nM	50 nM + 50 nM	100 nM + 100 nM		
**Rapamycin + MG-132 + CQ.**			RAP 50 nM + MG-132 50 nM + CQ 50 μ			
**Rapamycin + MG-132 + 3-MA**			RAP 50 nM + MG-132 50 nM + 10 mM 3-MA			
**Resveratrol (RES)**	10 μM	50 μM	10 μM	50 μM	10 μM	50 μM
**Resveratrol + CQ**			RES 10 μM + 50 μM CQ			
**Resveratrol + 3-MA**			RES 10 μM + 10 mM 3-MA			
**Resveratrol + Rapamycin**	10 μM + 50 nM	50 μM + 100 nM	10 μM + 50nM	50 μM + 100nM	10 μM + 50 nM	50 μM + 100 nM

## Supplementary Materials

Supplementary materials can be found at https://www.mdpi.com/1422-0067/21/3/813/s1.
