# Supplementary material for: Resveratrol as Inducer of Autophagy, Pro-Survival, and Anti-Inflammatory Stimuli in Cultured Human RPE Cells
Source: Int J Mol Sci. 2020 Jan 27;21(3):813. doi: 10.3390/ijms21030813 (PMC7036848; doi:10.3390/ijms21030813)
Supplement: Supplementary file 1 [file ijms-21-00813-s001.zip › Supplementary Table 1.pdf]

Supplementary Table S1. List of analytes on the protein array.

Coordinate	Analyte/Control	Alternate Nomenclature
A1,A2	Reference Spots	RS
A3,A4	Adiponectin	Acrp30
A5,A6	Aggrecan	Aggrecan 1
A7,A8	Angiogenin	
A9,A10	Angiopoietin-1	Ang-1, ANGPT1
A11,A12	Angiopoietin-2	Ang-2, ANGPT2
A13,A14	BAFF	BLyS, TNFSF13B
A15,A16	BDNF	Brain-derived Neurotrophic Factor
A17,A18	Complement Component C5/C5a	C5/C5a
A19,A20	CD14	TNFRSF8
A21, A22	CD30	
A23,A24	Reference Spots	RS
B3, B4	CD40 ligand	CD40L, TNFSF5, CD154, TRAP
B5, B6	Chitinase 3-like 1	CHI3L1, YKL-40
B7, B8	Complement Factor D	Adipsin, CFD
B9, B10	C-Reactive Protein	CRP, Factor
B11, B12	Cripto-1	Teratocarcinoma-derived Growth
B13, B14	Cystatin C	CST3, ARMD11
B15, B16	Dkk-1	Dickkopf-1
B17, B18	DPPIV	CD26, DPP4, Dipeptidyl-peptidase
B19, B20	EGF	Epidermal Growth Factor
B21, B22	EMMPRIN	CD147, Basigin IV,
C3, C4	ENA-78	CXCL5
C5, C6	Endoglin	CD105, ENG
C7, C8	Fas Ligand	TNFSF6, CD178, CD95L
C9, C10	FGF basic	FGF-2
C11, C12	FGF-7	KGF
C13, C14	FGF-19	
C15, C16	Flt-3 Ligand	FLT3LG
C17, C18	G-CSF	CSF3
C19, C20	GDF-15	MIC-1
C21, C22	GM-CSF	CSF2
D1, D2	GRO-α	CXCL1, MSGA-α
D3, D4	Growth Hormone	GH, Somatotropin
D5, D6	HGF	Scatter Factor, SF
D7, D8	ICAM-1	CD54
D9, D10	IFN-γ	IFNG
D11, D12	IGFBP-2	
D13, D14	IGFBP-3	
D15, D16	IL-1α	IL-1F1
D17, D18	IL-1β	IL-1F2
D19, D20	IL-1ra	IL-1F3
D21, D22	IL-2	
D23, D24	IL-3	

E1, E2	IL-4	
E3, E4	IL-5	
E5, E6	IL-6	
E7, E8	IL-8	CXCL8
E9, E10	IL-10	
E11, E12	IL-11	
E13, E14	IL-12, p70	
E15, E16	IL-13	
E17, E18	IL-15	
E19, E20	IL-16	
E21, E22	IL-17A	IL-17, CTLA8
E23, E24	IL-18 Bpa	
F1, F2	IL-19	
F3, F4	IL-22	IL-TIF
F5, F6	IL-23	IL-23A, SGRF
F7, F8	IL-24	C49A, FISP, MDA-7, MOB-5, ST16
F9, F10	IL-27	
F11, F12	IL-31	
F13, F14	IL-32α/β/γ	
F15, F16	IL-33	C9orf26, DVS27, NF-HEV
F17, F18	IL-34	C16orf77
F19, F20	IP-10	CXCL10
F21, F22	I-TAC	CXCL11, SCYB9B
F23, F24	Kallikrein 3	PSA, KLK3
G1, G2	Leptin	OB
G3, G4	LIF	
G5, G6	Lipocalin-2	NGAL, LCN2, Siderocalin
G7, G8	MCP-1	CCL2, MCAF
G9, G10	MCP-3	CCL7, MARC
G11, 12	M-CSF	CSF1
G13, 14	MIF	
G15, 16	MIG	CXCL9
G17, 18	MIP-1α/MIP-1β	CCL3/CCL4
G19, 20	MIP-3α	CCL20, Exodus-1, LARC
G21, 22	MIP-3β	CCL19, ELC
G23, G24	MMP-9	CLG4B, Gelatinase B
H1, H2	Myeloperoxidase	MPO, Lactoperoxidase
H3, H4	Osteopontin	OPN
H5, H6	PDGF-AA	
H7, H8	PDGF-AB/BB	
H9, H10	Pentraxin-3	PTX3, TSG-14
H11, H12	PF4	CXCL4
H13, H14	RAGE	
H15, H16	RANTES	CCL5
H17, H18	RBP4	
H19, H20	Relaxin-2	RLN2, RLXH2
H21, H22	Resistin	ADSF, FIZZ3, RETN
H23, H24	SDF-1α	CXCL12, PBSF

I1, I2	Serpin E1	PAI-I, PAI-1, Nexin
I3,I4	SHBG	ABP
I5, I6	ST2	IL-1, R4, IL1RL1, ST2L
I7, I8	TARC	CCL17
I9, I10	TFF3	ITF, TFI
I11, I12	TfR	CD71, TFR1, TFRC, TRFR
I13, I14	TGF-α	TGFA
I15, I16	Thrombospondin-1	THBS1, TSP-1
I17, I18	TNF-alpha	TNFSF1A
I19, I20	uPAR	PLAUR
I21, I22	VEGF	BEGFA
J1, J2	Reference Spots	RS
J5, J6	Vitamin D BP	VDB, DBP, VDBP
J23, J24	Negative Controls	Control (-)
